# Theoretical Study of the Effect of π-Bridge on Optical and Electronic Properties of Carbazole-Based Sensitizers for DSSCs

**DOI:** 10.3390/molecules25163670

**Published:** 2020-08-12

**Authors:** Tomás Delgado-Montiel, Jesús Baldenebro-López, Rody Soto-Rojo, Daniel Glossman-Mitnik

**Affiliations:** 1 Facultad de Ingeniería Mochis, Universidad Autónoma de Sinaloa, Prol. Ángel Flores y Fuente de Poseidón, S/N, Los Mochis 81223, Sinaloa, Mexico; tomas.delgado@uas.edu.mx (T.D.-M.); rody.soto@uas.edu.mx (R.S.-R.); 2 Laboratorio Virtual NANOCOSMOS, Departamento de Medio Ambiente y Energía, Centro de Investigación en Materiales Avanzados, Miguel de Cervantes 120, Complejo Industrial Chihuahua, Chihuahua 31136, Chihuahua, Mexico; daniel.glossman@cimav.edu.mx

**Keywords:** carbazole, DFT, DSSC, chemical hardness, free energy of electron injection, thiophene group, π-bridge

## Abstract

Eight novel metal-free organic sensitizers were proposed for dye-sensitized solar cells (DSSCs), theoretically calculated and studied via density functional theory with D-π-A structure. These proposals were formed to study the effect of novel π-bridges, using carbazole as the donor group and cyanoacrylic acid as the anchorage group. Through the M06/6-31G(d) level of theory, ground state geometry optimization, vibrational frequencies, the highest occupied molecular orbital, the lowest unoccupied molecular orbital, and their energy levels were calculated. Further, chemical reactivity parameters were obtained and analyzed, such as chemical hardness (η), electrophilicity index (ω), electroaccepting power (ω+) and electrodonating power (ω-). Free energy of electron injection (ΔGinj) and light-harvesting efficiency (LHE) also were calculated and discussed. On the other hand, absorption wavelengths, oscillator strengths, and electron transitions were calculated through time-dependent density functional theory with the M06-2X/6-31G(d) level of theory. In conclusion, the inclusion of thiophene groups and the Si heteroatom in the π-bridge improved charge transfer, chemical stability, and other optoelectronic properties of carbazole-based dyes.

## 1. Introduction

**Figure 1:**
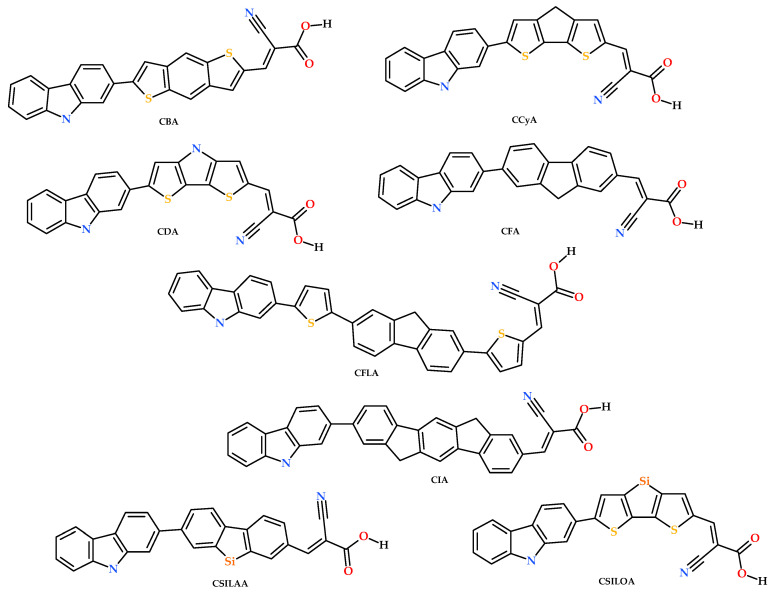
Molecular structure of carbazole-based dyes with different conjugated π-bridges.

Modernity depends to a large extent on the production of electric power. Currently, 66% of the world’s production depends on fossil fuel supply [1,2]. For this and other environmental reasons, comes the need to make clean energy from renewable energy [3]. Dye-sensitized solar cells (DSSCs) are a novel proposal to create clean and environmentally friendly energy [4]. In their beginnings, DSSCs used ruthenium II-based sensitizers and later, metal-free organic sensitizers were utilized, making DSSC a promising alternative. These devices consist mainly of four components for their operation: a sensitizer, semiconductor oxide (TiO_2_), a counter electrode (Pt), and an electrolyte (*I*^−^*/I*^−^_3_). The sensitizer has been a key component to improve the device’s efficiency [5,6]. There are mainly two types of sensitizers: (i) metal complexes, such as polypyridyl Ru (II) with which DSSCs reached a photoelectric conversion efficiency of approximately 12% [7]. However, Ru (II) based sensitizers have the disadvantage of requiring a very complex purification that raises its cost, and besides, its residues have a considerable toxicity grade. (ii) metal-free organic sensitizers with which DSSCs reached photoelectric efficiency of 14.5% [8]. They have the advantage of being low cost and are easy to manufacture, having large absorption coefficients, a feasible molecular design, prominent optoelectronic properties, and environmental friendliness [9]. Metal-free organic sensitizers that have generated higher efficiencies in DSSCs have a donor-π-bridge-acceptor (D-π-A) structure. These sensitizers have a photoinduced intramolecular charge transfer from the donor area to the acceptor through the π-bridge, also known as the “spacer”. Through a strong anchorage, electrons are injected to the semiconductor’s conduction band, causing an electron flow available to an electrical circuit, through which they return to the DSSC’s counter electrode that is immersed in an electrolyte responsible for regenerating the oxidized sensitizer [10]. Charge transfer through the π-bridge plays an essential role in DSSC efficiency [11]. Metal-free organic sensitizers have used a variety of organic electron donor compounds presenting an excellent performance, due to the ability to excite its electrons [12], such as coumarin [13], carbazole [14], indoline [15,16], phenothiazine [17], and triphenylamine [18,19]. The use of thiophene [20], benzene [21], dioxythiophene [22], and benzothiadiazole [23] on the π-bridge have been studied extensively. Cyanoacrylic acid and alkoxysilane [24] have been widely used as anchorage groups with excellent results [8]. Based on the above, methodologies have been made to study the four main components and to develop highly efficient DSSCs. One of the most effective techniques to make it is the modification of metal-free organic sensitizers, mainly by varying the chemical groups in the donor moiety, π-bridge and anchorage moiety [25]. In relation to donor moiety, carbazole has been used, for its donor capacity [26,27,28,29], in the development of diodes [30], organic field-effect transistors [31], solar cells [32], sensors [33] and OLEDs [34]. Carbazole has good electron delocalization [35] and it is planar with an electron-rich aromatic heterocycle [36]. On the other hand, cyanoacrylic acid is the most reported acceptor in literature, for it has good electron attraction properties [37]. This generates a good charge transfer between the sensitizer and the semiconductor conduction band [38]. In this work, we investigate a series of D-π-A organic dyes to study the effect of the π-bridge and its importance in the metal-free organic sensitizer, maintaining donor and acceptor units as carbazole and cyanoacrylic acid, respectively. Thereby, eight different π-bridge units were proposed to study its influence on the optoelectronic properties of the sensitizer and improve the efficiency of the DSSC. These π-bridges units are: benzodithiophene [39], cyclopentadithiophene [40], dithienopyrrole [41], fluorene [42], fluorenebisthiophene, indenofluoreno, silafluorene and silolodithiophene [42]. In this order, the dyes were named: CBA, CCyA, CDA, CFA, CFLA, CIA, CSILAA and CSILOA, which are shown in Figure 1. This study aims to have a diversity of chemical groups in the π-bridge, such as: (a) the presence of heteroatoms; from CCyA structure a carbon atom (C) is replaced by a nitrogen atom (N) named now CDA. Llater, the substitution of the same atom by a silicon atom (Si) is named now CSILOA, as well as from CFA a carbon atom is replaced by a silicon atom obtaining CSILAA; (b) the increase in the π bonds (conjugation) as is the case of CFA and CIA; (c) the presence of the thiophene group. Most of these groups have not been extensively studied as a π-bridge in a sensitizer for application in DSSC.

## 2. Results

### 2.1. Molecular Structure of Dyes

Optimization of the geometry of the fundamental state of the sensitizers was carried out in vacuum with the M06/6-31G(d) level of theory. First, a conformational analysis was carried out followed by dye optimization to find the most stable geometry. The optimized structures correspond to an energy minimum which were verified by calculating vibrational frequencies (not imaginary frequencies). XYZ coordinates of the optimized molecular structure carbazole-based dyes are displayed in the supplementary material. From the optimized structures, the geometric parameters were analyzed, such as bond lengths and dihedral angles; the most important ones are shown in Table 1. The dihedral angle between the donor unit (D) and the π-bridge unit (π), which was named (φD-π), as well as the dihedral angle between the π-bridge unit (π) and the acceptor unit (A), which was called (φπ-A). About the dihedral angle (φD-π), the sensitizers that have a thiophene group in the π-bridge linked to the carbazole unit have smaller dihedral angles, such as CBA of −24.56°, CCyA of −25.87°, CFLA of 26.09°, CSILOA of 26.74° and CDA of 26.92°. In comparison, the higher dihedral angles correspond to the phenyl group linked to the carbazole unit, such as CSILAA of −36.77°, CIA of 36.97°, and CFA of −37.33°. This is due to the phenyl group, which causes a steric repulsion between the carbazole and phenyl’s hydrogens. It is important to note that the dihedral angle between the donor (D) and π-bright (π) moieties plays an important role in intramolecular charge transfer [42,43,44]. On the other hand, the dihedral angle (φπ-A) of all sensitizers presents high planarity. It supports the charge transfer from π-bridge to the acceptor; therefore, the electron injection from sensitizer to the TiO_2_ conduction band. According to planarity, the best sensitizers are CBA, CCyA, CFLA, CSILOA, and CDA. Note that these sensitizers have a thiophene group linked to the carbazole donor unit.

### 2.2. Frontier Molecular Orbitals

**Figure 2:**
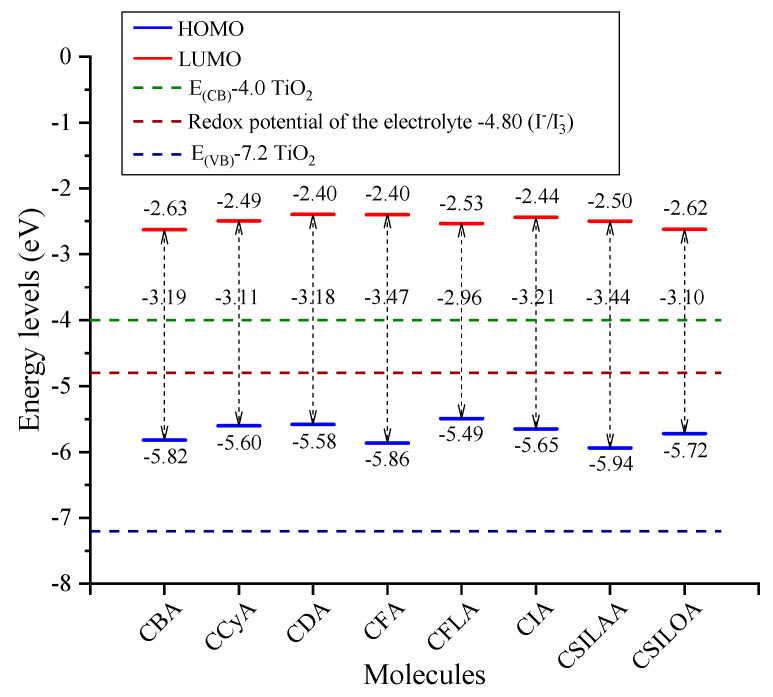
Highest occupied molecular orbital (HOMO) and lowest unoccupied molecular orbital (LUMO) energy levels of carbazole-based dyes calculatedwith the M06/6-31G(d) level of theory.

Frontier molecular orbitals energy levels were estimated with the M06/6-31G(d) level of theory. The energy levels obtained from both the highest occupied molecular orbital (HOMO) and the lowest unoccupied molecular orbital (LUMO) show that all the molecular systems have the characteristics required by a sensitizer and can be used in a DSSC. In the operation of the DSSC, under solar irradiation, the electron of the sensitizer is excited from HOMO to LUMO. The LUMO level must be above the TiO_2_ conduction band so that the electronic injection can be carried out from sensitizer to the semiconductor conduction band. The HOMO must be below the electrolyte (*I*^−^/*I*^−^_3_) redox potential to regenerate the oxidized sensitizer; this is shown in Figure 2. The CCyA, CDA, and CSILOA have a similar structure; the difference is that they have a different atom in the π-bridge. For example, CCyA has a cyclopentadithiophene in the π-bridge, in CDA it changes the atom from carbon to nitrogen located in the pentane ring, and in CSILOA it changes to silicon. In these three molecules, the HOMO-LUMO gap was different, although the conjugation of bonds does not change. The values decrease from CDA (N) with −3.18 eV, to CCyA (C) with −3.11 eV and to CSILOA (Si) with −3.10 eV. Of the after-mentioned, the lowest HOMO-LUMO gap was obtained for the inclusion of Si heteroatom. On the other hand, from the CFA, which has a fluorene as π-bridge, it was modified to a silafluorene and it was named CSILAA. Further, CFA was also modified by introducing a thiophene group at each side of the fluorene, named CFLA. It can be seen how the gap is reduced from −3.47 eV for CFA to −3.44 eV for CSILAA and to −2.96 eV for CFLA. Additionally, the CIA has a gap of −3.21 eV with an extended conjugation of bonds for using indenofluorene in the π-bridge, and CBA has a gap of −3.19 eV for using a benzobistiophene in the π-bridge. According to the lower value of the energy gap, the best sensitizers are CFLA, CSILOA, CCyA, CDA, and CBA, in this order. It found that, the higher the conjugation of bonds, the lower the HOMO-LUMO gap, and the use of heteroatoms combined with the above decreases it.

The HOMO and LUMO energy levels are key elements to predict intermolecular charge transfer. HOMO is related to the donor capacity of the sensitizer [45]. The energy difference between the electrolyte redox potential and the HOMO energy level is an important value since it determines the ease with which the sensitizer can be regenerated [46]. The donor moiety of the sensitizer can be modified with electron donor character groups and it increases the HOMO energy level, but it will also increase the LUMO energy value [47]. Thus, the driving force of electron injection increases (energy difference between the LUMO and the TiO_2_ conduction band), causing a decrease in electronic injection [46]. In this work, the driving force of electron injection increases as follows: CBA of −2.63 eV, CSILOA of −2.62 eV, CFLA of −2.53 eV, CSILAA of −2.50 eV and CCyA of −2.49 eV. Meanwhile, the sensitizers that could be more easily regenerated are CFLA of −5.49 eV, CDA of −5.58 eV, CCyA of −5.60 eV and CIA of −5.65 eV. The CFLA molecule presents the smallest HOMO-LUMO gap with a value of the HOMO energy level closer to the electrolyte redox potential, ensuring a better electronic regeneration of the dye. This parameter is relevant to improve the conversion efficiency [48]. On the other hand, CBA and CSILOA systems are those that present the LUMO energy values closest to the TiO_2_ conduction band, ensuring a better electronic injection to the semiconductor. However, these present a higher HOMO-LUMO gap than CFLA. Therefore, CFLA, CBA and CSILOA are the best sensitizers according to HOMO-LUMO energy levels.

**Figure 3:**
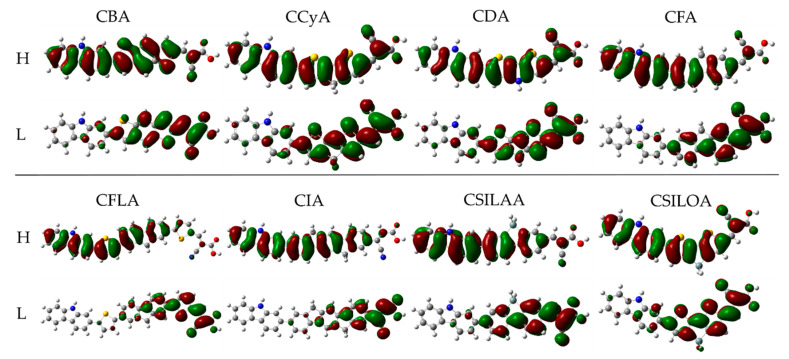
Density of HOMO and LUMO frontier molecular orbitals of the carbazole-based dyes at M06/6-31G(d) level of theory.

Figure 3 shows the location of HOMO and LUMO frontier molecular orbital density of the eight proposed structures. The HOMO orbital in the CBA, CCyA, CDA, and CSILOA sensitizers is distributed on the whole molecule, while the HOMO in the CFA, CFLA, CIA, and CSILAA sensitizers is located mainly on the donor unit and the π-bridge. On the other hand, for all the molecules, the LUMO is located on the π-bridge and the acceptor group. Better charge separation is observed in the molecular systems with a larger π-bridge, CFLA and CIA.

### 2.3. Ultraviolet-Visible Absorption Spectra

**Figure 4:**
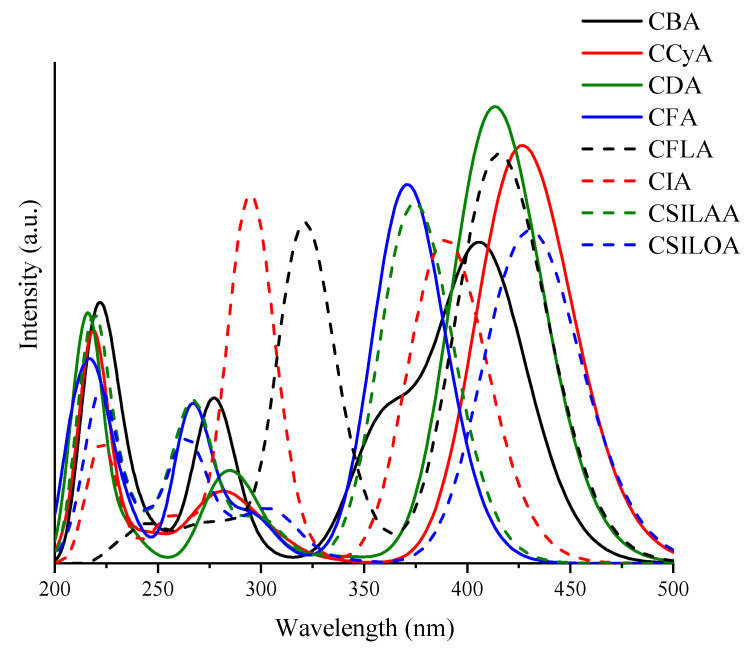
Ultraviolet Visible (UV-Vis) absorption spectra of carbazole-based dyes with the M06-2X/6-31G(d) level of theory.

The Ultraviolet Visible (UV-Vis) absorption spectra of eight molecules was calculated through the M06-2X/6-31G(d) level of theory. The solvation model used was the Integral Equation Formulation Polarizable Continuous Model (IEF-PCM), in the presence of chloroform. The molecules of interest are those that have the maximum absorption wavelength (λmax) with the greater bathochromic shift due to the lower energy requirement to carry out electron excitation using incident sunlight. Further, it is desirable that the electron transition of the λmax band corresponds meanly to HOMO to LUMO transition (H→L) and that this has the highest intensity, which is related to oscillator strength (*f*). In Figure 4, it can be observed that five sensitizers have maximum absorption bands in the visible region. Three sensitizers have maximum absorption bands in the ultraviolet region. CSILOA exhibits the maximum absorption wavelength (λmax) with more bathochromic displacement at 430 nm. CCyA presents a similar λ_max_ at 427 nm with a higher intensity of the band. Next, according to λ_max_, CFLA at 416 nm, CDA at 414 nm and CBA at 407 nm. CIA, CSILAA, and CFA have a λ_max_ lower than 400 nm, mainly, on the ultraviolet region. Further, it can be observed that the structures with λ_max_ in the visible region are those that contain thiophene groups in the π-bridge linked to the donor unit. Furthermore, these present a high HOMO-LUMO transition and high oscillator strength related to λ_max_, as observed in Table 2. For example, CDA and CCyA have the highest H→L transitions and the highest oscillator strength (*f*), H→L (91%) with *f* = 1.638 for CDA, and H→L (90%) with *f* = 1.499 for CCyA. On the other hand, the molecules without thiophene group in the π-bridge, have a low or no H→L transition, though with a high *f* value. For example, CFA with fluorene in the π-bridge has a H→L transition of 26% with *f* = 1.358, CSILAA with silafluorene does not present H→L transition, and CIA with indenofluorene (similar to fluorene but larger) increases the H→L transition to 71% with *f* = 1.160. According to λ_max_, H→L transitions and oscillator strength, the best sensitizers can be CSILOA, CCyA, CDA, and CFLA. CSILOA, CCyA, and CDA have the same structure and are different for the atom located in the pentane ring of the cyclopentadithiophene, being Si for CSILOA, C for CCyA, N for CDA. Absorption spectra calculated with the CAM-B3LYP functional and the 6-31G(d) basis set are included in Figure S1 (in the supplementary materials). The analysis confirms the absorption bands’ behavior with a functional comprising of a Hartree-Fock exchange interaction at short-range and long-range.

### 2.4. Chemical Reactivity Parameters

**Figure 5:**
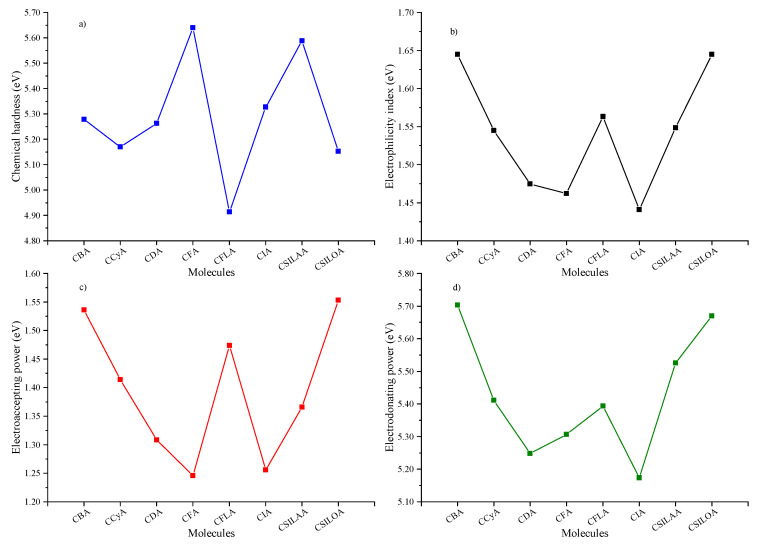
(**a**) Chemical hardness, (**b**) electrophilicity index, (**c**) electroaccepting power, and (**d**) electrodonating power of the molecular systems.

Chemical hardness describes the resistance of molecules to intramolecular charge transfer; therefore, the lower the chemical hardness, the higher the intramolecular charge transfer [49,50,51]. Chemical hardness has been related to the efficiency of DSSC [52,53,54]; the lower the chemical hardness, the higher the conversion efficiency. It may be a first reference parameter to choose the most efficient sensitizer in a DSSC from a group of many molecules. Through chemical hardness, the π-bridge effect about intramolecular charge transfer can be explained. Regarding the discussion carried out so far, the eight sensitizers can be grouped in two: (1) Sensitizers with a thiophene group in the π-bridge linked to donor unit: CBA, CCyA, CDA, CFLA, and CSILOA; (2) Sensitizers without a thiophene group in the π-bridge: CFA, CIA, and CSILAA. According to Figure 5a and Table 3, it can be observed as follows: in group 1, these are molecules with lower chemical hardness. CFLA has the lowest chemical hardness (4.91 eV), which has a higher π bonds conjugation, two thiophene groups and the lowest HOMO-LUMO gap, followed by CSILOA (5.15 eV), CCyA (5.17 eV), CDA (5.26 eV), and CBA (5.28 eV). Note that the charge transfer decreases from CSILOA (Si), CCyA (C), to CDA (N), due to the change of the atom in the pentane ring. In group 2, these molecules have higher chemical hardnesses than group 1. CFA with fluorene in the π-bridge has a chemical hardness of 5.64 eV. CSILAA with a Si atom in the pentane ring of the fluorene, as silafluorene, decreases the chemical hardness to 5.59 eV. CIA’s fluorene changes to indenofluorene, which has a higher π bonds conjugation, it decreases the chemical hardness to 5.33 eV. All sensitizers have a congruence of chemical hardness with the HOMO-LUMO gap. The lower the HOMO-LUMO gap, the lower the chemical hardness.

The electrophilicity index represents the stability of the sensitizers in the presence of external charges to the system [55,56]. In Figure 5b and Table 3, the molecules with higher stability are CBA (1.64 eV) and CSILOA (1.64 eV). They are followed by CFLA (1.56 eV), CSILAA (1.55), and CCyA (1.54 eV). Finally, with the lowest stability are CDA (1.47 eV), CFA (1.46 eV), and CIA (1.44 eV). It can be observed that the inclusion of the Si heteroatom in the sensitizer increases the electrophilicity index. For example, this occurs from CCyA (1.54 eV) to CSILOA (1.64 eV), and from CFA (1.46 eV) to CSILAA (1.55 eV). In the CDA, CCyA and CSILOA molecules can be observed as a tendency to increase the electrophilicity index in the order of the N, C, and Si atom. Note that later, this tendency is the same for electroaccepting power and occurs inversely for electrodonating power.

Electroaccepting power indicates the acceptor character of the molecule. The higher the electroaccepting power value, the higher the ability to accept electrons [55]. In Figure 5c and Table 3, it can be observed that the sensitizers with the highest electroaccepting power are CSILOA (1.55 eV) and CBA (1.54 eV). In a medium level, it is CFLA (1.47 eV), CCyA (1.41 eV), and CSILAA (1.37 eV). These are followed by the sensitizers with the lowest electroaccepting power, which are CDA (1.31 eV), CIA (1.26 eV), and CFA (1.25 eV). Electrodonating power indicates the ability of the molecules to donate electrons; in this case, the lower the electrodonating power value, the higher the donor character of the sensitizer [55]. In Figure 5d and Table 3, it can be observed that CIA (5.17 eV), CDA (5.25 eV), and CFA (5,31 eV) are the sensitizers that have a higher donor character. These are followed by CFLA (5.39 eV), CCyA (5.17 eV), CSILAA (5.53 eV), CSILOA (5.67 eV), and CBA (5.70 eV). Table S1 (supplementary material) shows the chemical reactivity parameters calculated with the CAM-B3LYP functional and the 6-31G(d) basis set. The analysis confirms the behavior of all values with a range-separated functional. According to both calculation methods, the difference between the values does not exceed 0.69 eV for the chemical hardness, 0.15 eV for the electrophilicity index, 0.24 eV for the electrodonating power, and 0.28 eV for the electroaccepting power.

Low chemical hardness and high electroaccepting power have been related to a better short-circuit current density (J_SC_); at the same time, this is an excellent property of photoelectric conversion efficiency [55,57]. As the electrophilicity index has the same tendency as the electroaccepting power, it can be related to J_SC_ [16]. In selecting a sensitizer with the best properties, importance is given to the chemical hardness parameter without neglecting the other three parameters. The aim is to have colorants with higher electrophilicity index values and electroaccepting power, but with lower electrodonating power values. Note that these values are opposed; therefore, it has been considered to choose average values of these three parameters, looking for an equilibrium between electroaccepting and electrodonating powers [52]. Considering the above, the best molecules are CFLA, CSILOA, and CCyA.

**Figure 6:**
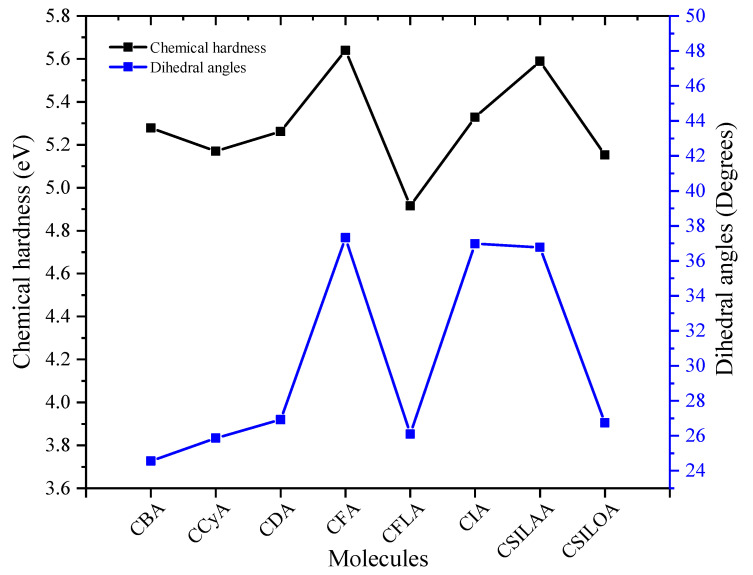
Comparison between chemical hardness (D-π) dihedral angles of the carbazole-based dyes.

Figure 6 shows that chemical hardness is directly related to the D-π dihedral angle (previously described), which is, the smaller the dihedral angle, the lower the chemical hardness. CBA, CCyA, CFLA, CSILOA, and CDA sensitizers are systems that have a thiophene group anchored to carbazole; this generates a lower dihedral angle and consequently, lower chemical hardness. Moreover, CSILAA, CIA, and CFA are systems that have a phenyl group anchored to carbazole. The closeness between the phenyl group of the π-bridge and the phenyl group of the carbazole induces a steric hindrance for the hydrogen atoms, hence, a higher D-π dihedral angle. On the other hand, planarity has been related to the aggregation of sensitizers [58]. This affects the efficiency of the DSSC, but it can be resolved by including aliphatic chains to avoid the π-π stacking among sensitizers. Further, planarity promotes a better intramolecular charge transfer (ICT) [59,60], which improves the conversion efficiency of the DSSC.

### 2.5. Free Energy of Electron Injection

One of the key factors of the short-circuit current density (J_SC_) is the electron injection efficiency, which is influenced by the free energy of electron injection (∆G_inj_); this is the difference between the oxidation potential energy of the excited state (Eox^dye^*) and the reduction potential energy of the TiO_2_ conduction band (E_CB_ = 4.0 eV). Successively, Eox^dye^* is the difference between the ground-state oxidation potential (Eox^dye^ = HOMO level) of the dye and the absorption energy associated with λ_max_ (vertical excitation energy ∆E) [26]. The absolute values of ∆G_inj_ for all dyes are much greater than 0.2 eV (Table 4), and according to the literature [61], it can be predicted that theses dyes have enough driving force for the fast injection of excited electrons from dye to TiO_2_ E_CB_. The ∆G_inj_ oscillate from 1.53 to 1.16 eV, which is high enough to guarantee an efficient electron injection. Besides, if ∆G_inj_ is too large, it may introduce energy redundancy and result in a smaller open-circuit voltage (V_OC_) and large thermalization losses [62]. Furthermore, the lower ∆G_inj_ is reflected in the higher electron injection from sensitizer to TiO_2_ conduction band. Then, the electron injection (in relation to ∆G_inj_) decreases in this order: CSILOA (1.16) < CBA (1.23) < CCyA (1.37) < CSILAA (1.37) < CDA (1.42) < CFLA (1.49) < CFA (1.51) < CIA (1.53). The results reflect that all the dyes have good electron injection efficiencies, with the best being CSILOA, CBA, CCyA, and CSILAA.

On the other hand, the oscillator strength (*f*) parameter is related to light-harvesting efficiency (LHE) [63,64,65], and its higher values indicate that short-circuit photocurrent (J_SC_) can be increased [66]. According to LHE, the best dyes could be CDA with 0.98, CFLA with 0.97, and CCyA with 0.97. These sensitizers also have been presenting good properties in the rest of the parameters discussed. It is important to note that all sensitizers present an LHE very close among them. It should be noted that LHE only contemplates the maximum absorption wavelength (λ_max_) and does not represent all other bands with a high *f* that also contribute to the absorption of the sensitizer, and so they should be considered in light harvesting.

## 3. Methods

### 3.1. Computational Details

All the optimization calculations of the ground state structures were carried out in vacuum using the density functional theory (DFT) [67,68]. The energy levels of the highest occupied molecular orbital (HOMO) and the lowest unoccupied molecular orbital (LUMO) were calculated along with their electron density. The analysis of frequencies was carried out to confirm that there are no imaginary frequencies; namely, the geometry is in a global minimum. The calculation of the chemical reactivity parameters was obtained, using conceptual DFT, through the ionic and neutral energies of the molecules. The chemical reactivity parameters studied are chemical hardness (η) [69], electrodonating power (ω^−^), electroaccepting power (ω^+^) [55], and electrophilicity index (ω) [56]. All previous calculations were carried out through the M06 hybrid meta-GGA density functional [70] and the 6-31G(d) basis set [71,72]. The calculation of the UV-Vis spectra was performed through time-dependent density functional theory (TD-DFT) [73,74] with the M06-2X hybrid Meta-GGA density functional [70] and the 6-31G(d) basis set [71,72]. Non-equilibrium protocol [75,76] solved for 20 excited states was used considering chloroform as solvent. This effect was evaluated with the Integral Equational Formalism Polarizable Continuum Model (IEF-PCM) [77]. The UV-Vis spectra were processed with SWizard program [78]. Also, the free energy of electron injection (∆G_inj_) was obtained for all molecules, this being the difference between oxidation potential energy of the excited state (Eox^dye^*) and the reduction potential energy of TiO_2_ conduction band (E_CB_ = −4.0 eV) [26,79]. The light-harvesting efficiency (LHE) was obtained by LHE(λ_max_) = 1 − 10^−*f*^, where *f* is the oscillator strength associated to λ_max_ [80,81]. The prediction of the absorption spectra and the aforementioned chemical reactivity parameters were also performed with the CAM-B3LYP range-separate functional; the objective was to verify that there are no significant differences in the selection of the calculation methodology [82]. All calculations were performed using the Gaussian 09 Revision D0.1 program [83].

### 3.2. Theoretical Background

Ionization potential (*I*) is obtained from the difference between the energy of the base state of the system with the number of electrons N_0_ − 1 (cation energy) and the base state of the system with the number of electrons N_0_ (neutral state) [84].

(1) $$I=\left(E_{N_{0}-1}-E_{N_{0}}\right),$$

Electron affinity (*A*) is obtained from the difference of the ground state of the system with the number of electrons N_0_ and base system state with the number of electrons N_0_ + 1 (anion energy) [84].

(2) $$A=\left(E_{N_{0}}-E_{N_{0}+1}\right),$$

Ionization potential is the minimum energy needed to remove an electron from an atom when it is in a gaseous and electrically neutral state, and electron affinity is the energy released when a neutral gaseous atom in its ground state captures an electron.

Molecular chemical hardness is defined as the resistance to charge transfer, which is obtained as [85]:

(3) n=(I−A),

Electrophilicity index was proposed by Parr [63], defined as the change in the energy of a molecule immersed in an environment saturated with electrons, which can be calculated with [55,56]:

(4) $$\omega =\frac{{\left(I+A\right)}^{2}}{8\left(I-A\right)},$$

Electrodonating power is the propensity to donate charge; smaller values imply a larger capability to donate charge [55]:

(5) $$\omega^{-}=\frac{{\left(3I+A\right)}^{2}}{16\left(I-A\right)}\;,$$

Electroaccepting power is the ability of the chemical species to accept charge and is given by [55]:

(6) $$\omega^{+}=\frac{{\left(I+3A\right)}^{2}}{16\left(I-A\right)},$$

The high light to electricity conversion efficiency is related to high values of the short-circuit current density (J_SC_). At the same time, J_SC_ is directly related to the electron injection efficiency, which is inversely proportional to the free energy of electron injection (∆G_inj_); smaller ∆G_inj_ values are expected to have higher J_SC_ values. ∆G_inj_ is expressed as [26]:

(7) $$\Delta {G\;}_{\mathrm{inj}}={\;\mathrm{Eox}}^{\mathrm{dye}*}-{\;E}_{\mathrm{CB}},$$

where Eox^dye^* is the oxidation potential energy excited state and E_CB_ is the reduction potential energy of TiO_2_ conduction band (E_CB_ = 4.0 eV). Eox^dye^* is expressed as [26]:

(8) $${\mathrm{Eox}}^{\mathrm{dye}*}={\;\mathrm{Eox}}^{\mathrm{dye}}-\Delta E,$$

where Eox^dye^ is the ground-state oxidation potential (HOMO level) of the dye and ∆E is the absorption energy associated with maximum absorption wavelength (λ_max_ in eV). The absolute values of ∆G_inj_ for all dyes are much greater than 0.2 eV (Table 4), and, according to the literature [61], it can be predicted that theses dyes have enough driving force for the fast injection of excited electrons from dye to TiO_2_ E_CB_.

J_SC_ is the integral of the short-circuit photocurrent density. This depends on the absorption coefficient of the dye and the interaction between the dye and the nanocrystalline TiO_2_ surface. It can be determined using the following equation [86]:

(9) $$J_{\mathrm{sc}}=\int_{\lambda }\mathrm{LHE}\;\left(\lambda \right)\Phi^{inject}\eta^{collect}d\lambda ,$$

where ƞ*^collect^* is the efficiency of the electron collection, which can be assumed to be constant for systems that only differ in the dye employed, the parameter Φ*^inject^* is the quantum yield of electron injection, while LHE(λ) is the light-harvesting efficiency at a given wavelength, which can be calculated using the following expression [87,88].

Short-circuit current density (J_SC_) also is directly related to the light-harvesting efficiency (LHE), which is expressed as:

(10) $$\mathrm{LHE}\left(\lambda_{\max}\right)=1-10^{-f},$$

where *f* is the oscillator strength associated to maximum absorption wavelength (λ_max_) [80,81].

## 4. Conclusions

In this work, eight novel carbazole donor-based sensitizers were designed and studied using novel π-bridges. The effect of the π-bridge was analyzed with different chemical groups. All molecules can be considered as sensitizers according to HOMO and LUMO levels of energy. Five sensitizers present maximum absorption wavelengths in the visible region. The inclusion of thiophene groups and the Si heteroatom in the π-bridge improves the optoelectronic properties, such as bathochromic displacement, reduction of HOMO-LUMO gap, intramolecular charge transfer, and chemical stability. Analyzing overall all properties, the best sensitizers with the potential to obtain highly efficient DSSCs are CFLA, CSILOA, and CCyA. The synthesis and application of these sensitizers on the DSSC is recommended to the experimental scientific community.

## Figures and Tables

**Table 1:** Summary of bond lengths (in angstroms, Å) and dihedral angles (in degrees, °) between the donor unit, π-bright, and acceptor unit of the optimized structures with the M06/6-31G(d) level of calculation.

Molecule	Donor Unit (D) (φD-π)	Acceptor Unit (A) (φπ-A)
CBA		
Dihedral angle	−24.6	−0.73
Bond length	1.46	1.42
CCyA		
Dihedral angle	−25.9	−0.04
Bond length	1.46	1.41
CDA		
Dihedral angle	26.9	0.09
Bond length	1.46	1.41
CFA		
Dihedral angle	−37.3	−0.62
Bond length	1.48	1.44
CFLA		
Dihedral angle	26.1	0.99
Bond length	1.46	1.42
CIA		
Dihedral angle	37.0	−21.29
Bond length	1.48	1.47
CSILAA		
Dihedral angle	−36.8	0.38
Bond length	1.47	1.44
CSILOA		
Dihedral angle	26.7	−0.11
Bond length	1.46	1.42

**Table 2:** Absorption wavelengths, vertical excitation energy (E), oscillator strengths (*f*), and the orbitals involved in the transitions of carbazole-based dyes with the functional M06-2X and the basis set 6-31G(d), using chloroform as solvent.

Molecule	λ_abs_ (nm)	E (eV)	*f*	Transitions H = HOMO, L = LUMO (%)
CBA	407	3.05	1.128	H →L (79%)
	360	3.44	0.509	H-2 → L (54%); H-3 → L (28%)
	277	4.47	0.508	H → L + 1 (71%)
CCyA	427	2.91	1.499	H → L (90%)
	284	4.36	0.189	H → L + 1 (74%)
	269	4.61	0.110	H-4 → L (37%); H-6 → L (31%)
CDA	414	3.00	1.638	H → L (91%)
	293	4.23	0.187	H-2 → L (48%); H → L+1 (22%)
	279	4.44	0.221	H → L + 1 (67%)
CFA	371	3.34	1.358	H-1 → L (49%); H → L (26%); H-2 → L (20%)
	266	4.66	0.448	H-1 → L + 1 (41%)
	211	5.88	0.321	H → L + 5 (30%)
CFLA	416	2.98	1.469	H → L (60%); H-2 → L (25%);
	341	3.64	0.147	H-2 → L (39%); H → L (31%) H-3 → L (24%)
	320	3.87	1.167	H → L + 1 (73%)
CIA	390	3.18	1.160	H → L (71%); H-2 → L (20%)
	295	4.20	1.253	H → L + 1 (77%)
	255	4.87	0.116	H-6 → L (30%)
CSILAA	375	3.31	1.291	H-1 → L (70%); H-2 → L (21%)
	268	4.62	0.365	H-1 → L + 1 (43%)
	220	5.64	0.536	H → L + 5 (25%)
CSILOA	430	2.88	1.194	H → L (86%)
	307	4.03	0.156	H-2 → L (74%)
	264	4.70	0.232	H → L + 2 (32%); H-4 → L (29%)

**Table 3:** Chemical reactivity parameters of carbazole-based dyes (in eV) obtained by density functional theory (DFT) conceptual at M06/6-31G(d) level of theory.

Molecules	η	ω	ω^−^	ω^+^
CBA	5.28	1.64	5.70	1.54
CCyA	5.17	1.54	5.41	1.41
CDA	5.26	1.47	5.25	1.31
CFA	5.64	1.46	5.31	1.25
CFLA	4.91	1.56	5.39	1.47
CIA	5.33	1.44	5.17	1.26
CSILAA	5.59	1.55	5.53	1.37
CSILOA	5.15	1.64	5.67	1.55

**Table 4:** Ground-state oxidation potential energy (Eox^dye^), absorption energy associated with λ_max_ (∆E), oxidation potential energy of the excited state (Eox^dye^*), free energy of electron injection (∆G_inj_), and light-harvesting efficiency (LHE).

Molecule	Eox^dye^ (eV)	∆E (eV)	Eox^dye^* (eV)	∆G_inj_ (eV)	LHE
CBA	5.82	3.05	2.77	1.23	0.93
CCyA	5.60	2.91	2.69	1.31	0.97
CDA	5.58	3.00	2.58	1.42	0.98
CFA	5.86	3.37	2.49	1.51	0.96
CFLA	5.49	2.98	2.51	1.49	0.97
CIA	5.65	3.18	2.47	1.53	0.93
CSILAA	5.94	3.31	2.63	1.37	0.95
CSILOA	5.72	2.88	2.84	1.16	0.94

## Supplementary Materials

The following are available online. Figure S1: UV-Vis absorption spectra of carbazole-based dyes with the CAM-B3LYP/6-31G(d) level of theory, Table S1: Chemical reactivity parameters of carbazole-based dyes (in eV) obtained by DFT conceptual at CAM-B3LYP/6-31G(d) level of theory.
