# Supplementary material for: Theoretical Study of the Effect of π-Bridge on Optical and Electronic Properties of Carbazole-Based Sensitizers for DSSCs
Source: Molecules. 2020 Aug 12;25(16):3670. doi: 10.3390/molecules25163670 (PMC7464466; doi:10.3390/molecules25163670)
Supplement: Supplementary file 1 [file molecules-25-03670-s001.pdf]

SUPPLEMENTARY MATERIAL

Theoretical Study of the Effect of π-Bridge on Optical and Electronic Properties of Carbazole-Based Sensitizers for DSSCs

Tomás Delgado-Montiel [1], Jesús Baldenebro-López [1,*], Rody Soto-Rojo [1] and Daniel Glossman-Mitnik [2]

1 Facultad de Ingeniería Mochis, Universidad Autónoma de Sinaloa, Prol. Ángel Flores y Fuente de Poseidón, S/N, Los Mochis 81223, Sinaloa, Mexico; tomas.delgado@uas.edu.mx (T.D.-M.); rody.soto@uas.edu.mx (R.S.-R.)

2 Laboratorio Virtual NANOCOSMOS, Departamento de Medio Ambiente y Energía, Centro de Investigación en Materiales Avanzados, Miguel de Cervantes 120, Complejo Industrial Chihuahua, Chihuahua 31136, Chihuahua, Mexico; daniel.glossman@cimav.edu.mx

* Correspondence: jesus.baldenebro@uas.edu.mx, Tel./Fax: +526688127641

TABLE OF CONTENTS

XYZ coordinates of the molecular structure of carbazole-based dyes **3**

Optimized Cartesian coordinates for the CBA molecular system 3

Optimized Cartesian coordinates for the CCyA molecular system 4

Optimized Cartesian coordinates for the CDA molecular system 5

Optimized Cartesian coordinates for the CFA molecular system 6

Optimized Cartesian coordinates for the CFLA molecular system 7

Optimized Cartesian coordinates for the CIA molecular system 8

Optimized Cartesian coordinates for the CSILAA molecular system 9

Optimized Cartesian coordinates for the CSILOA molecular system 10

Ultraviolet-Visible absorption spectra **11**

Chemical reactivity parameters **11**

XYZ coordinates of the molecular structure of carbazole-based dyes

Optimized Cartesian coordinates for the CBA molecular system

Geometrical parameters

Center Number	Coordinates (Angstroms) X	Y	Z
C	3.726307000	1.516630000	-0.467524000
C	3.186153000	0.260537000	-0.115733000
C	4.040400000	-0.794344000	0.217101000
C	5.409195000	-0.566749000	0.195201000
C	5.956157000	0.691868000	-0.147471000
C	5.091549000	1.735316000	-0.480525000
H	3.050908000	2.316815000	-0.763377000
H	3.639063000	-1.763647000	0.512992000
H	5.485772000	2.710785000	-0.762556000
C	7.652394000	-0.771763000	0.328855000
C	8.949741000	-1.244539000	0.501034000
C	9.991323000	-0.355656000	0.276194000
C	9.751040000	0.970747000	-0.109071000
C	8.455938000	1.434598000	-0.279801000
C	7.389210000	0.561037000	-0.062486000
H	9.141518000	-2.273891000	0.799851000
H	11.016937000	-0.697951000	0.402490000
H	10.591778000	1.640888000	-0.276290000
H	8.271859000	2.465722000	-0.579607000
N	6.446295000	-1.431825000	0.483170000
H	6.341175000	-2.399348000	0.749091000
C	1.738320000	0.079479000	-0.106521000
C	0.771547000	1.029315000	0.043246000
C	-0.560259000	0.517165000	0.000224000
C	-0.577286000	-0.895714000	-0.188138000
S	1.049819000	-1.532159000	-0.320417000
C	-1.764409000	1.214914000	0.124329000
C	-2.946323000	0.495805000	0.051187000
C	-2.959676000	-0.914508000	-0.142469000
C	-1.751273000	-1.616387000	-0.260539000
C	-4.283622000	-1.418514000	-0.190837000
C	-5.253729000	-0.456645000	-0.041086000
S	-4.563204000	1.155056000	0.171756000
H	0.995093000	2.079793000	0.215695000
H	-1.752160000	-2.695596000	-0.406013000
H	-1.764311000	2.293741000	0.271856000
H	-4.526359000	-2.469844000	-0.335388000
C	-6.646096000	-0.755894000	-0.065924000
C	-9.064471000	-0.571119000	-0.013695000
C	-7.630417000	1.461543000	0.289111000
N	-7.542826000	2.608496000	0.468313000
C	-7.726903000	0.057630000	0.071485000
H	-6.890712000	-1.809083000	-0.222638000
O	-9.259329000	-1.751989000	-0.191167000
O	-10.052569000	0.325098000	0.128455000
H	-10.884382000	-0.174814000	0.056877000

Optimized Cartesian coordinates for the CCyA molecular system

Geometrical parameters

Center Number	Coordinates (Angstroms) X	Y	Z
C	-6.351556000	0.359919000	0.127253000
C	-7.043140000	-0.808335000	-0.024948000
C	-6.403385000	-2.054594000	-0.265219000
C	-8.512347000	-0.747422000	0.073345000
H	-6.983349000	1.233596000	0.306432000
N	-5.852087000	-3.062070000	-0.461275000
O	-9.152247000	0.261311000	0.275267000
O	-9.082290000	-1.954210000	-0.086667000
H	-10.040646000	-1.809319000	-0.005016000
C	3.680147000	1.827242000	-0.454758000
C	2.824167000	0.764145000	-0.091871000
C	3.367628000	-0.484852000	0.225402000
C	4.745711000	-0.638105000	0.173222000
C	5.608758000	0.426709000	-0.176858000
C	5.053348000	1.667048000	-0.492927000
H	3.242304000	2.782804000	-0.737146000
H	2.727402000	-1.310341000	0.536687000
H	5.691890000	2.502062000	-0.778738000
C	6.851219000	-1.442715000	0.279834000
C	7.974571000	-2.246882000	0.446359000
C	9.216039000	-1.670212000	0.217986000
C	9.339227000	-0.328147000	-0.167503000
C	8.215478000	0.466802000	-0.333087000
C	6.954197000	-0.087272000	-0.109180000
H	7.883786000	-3.289668000	0.746264000
H	10.112780000	-2.274809000	0.341451000
H	10.328573000	0.092034000	-0.336999000
H	8.314169000	1.509589000	-0.632981000
C	1.384410000	0.987329000	-0.044323000
C	0.710287000	2.173946000	0.161645000
C	-0.686311000	2.011943000	0.159550000
C	-1.074919000	0.698335000	-0.050442000
S	0.270753000	-0.356268000	-0.262672000
H	1.226789000	3.113185000	0.343758000
C	-4.962760000	0.623962000	0.097256000
C	-4.407983000	1.890514000	0.283499000
C	-3.018407000	1.891681000	0.222136000
C	-2.500363000	0.618030000	-0.013026000
S	-3.703972000	-0.593409000	-0.163736000
H	-5.035021000	2.761809000	0.461593000
C	-1.895908000	2.887116000	0.348552000
H	-1.894288000	3.391185000	1.325883000
H	-1.956244000	3.679618000	-0.411571000
N	5.513108000	-1.753285000	0.443816000
H	5.153884000	-2.653344000	0.724943000

Optimized Cartesian coordinates for the CDA molecular system

Geometrical parameters

Center Number	Coordinates (Angstroms) X	Y	Z
C	3.642299000	1.777515000	0.468511000
C	2.834788000	0.681674000	0.092734000
C	3.431009000	-0.540771000	-0.231498000
C	4.814929000	-0.634236000	-0.176714000
C	5.629151000	0.464628000	0.184068000
C	5.020707000	1.676950000	0.509930000
H	3.161882000	2.709845000	0.759357000
H	2.825588000	-1.389832000	-0.549134000
H	5.622380000	2.535693000	0.805390000
C	6.955214000	-1.343397000	-0.283573000
C	8.114948000	-2.093572000	-0.452878000
C	9.327858000	-1.461957000	-0.215370000
C	9.388591000	-0.118924000	0.181462000
C	8.229302000	0.622098000	0.349514000
C	6.995843000	0.012061000	0.116364000
H	8.073731000	-3.137014000	-0.761191000
H	10.252027000	-2.023359000	-0.341010000
H	10.357575000	0.343878000	0.357650000
H	8.278506000	1.665888000	0.657960000
N	5.632363000	-1.712006000	-0.453911000
H	5.314119000	-2.625531000	-0.740946000
C	-2.927925000	1.713433000	-0.227867000
C	-2.476961000	0.400671000	0.013623000
C	-1.072898000	0.453588000	0.051119000
C	-0.704050000	1.787915000	-0.166761000
C	1.386989000	0.847756000	0.038463000
C	0.687739000	2.012582000	-0.175483000
H	-1.861869000	3.544071000	-0.513742000
H	1.170328000	2.966975000	-0.366215000
S	0.328357000	-0.544877000	0.267615000
S	-3.787479000	-0.714459000	0.157741000
C	-4.944597000	0.601491000	-0.110162000
C	-4.314969000	1.828253000	-0.297735000
H	-4.875315000	2.742351000	-0.477582000
N	-1.835373000	2.551980000	-0.336243000
C	-6.350556000	0.446197000	-0.139056000
C	-7.135956000	-0.659784000	0.018736000
C	-6.609481000	-1.957618000	0.262999000
C	-8.595298000	-0.470121000	-0.073906000
N	-6.154439000	-3.011287000	0.462312000
H	-6.912023000	1.366096000	-0.320215000
O	-9.144194000	0.590183000	-0.280381000
O	-9.269243000	-1.619952000	0.097872000
H	-10.211412000	-1.390928000	0.019809000

Optimized Cartesian coordinates for the CFA molecular system

Geometrical parameters

Center Number	X	Y	Z
C	3.882101000	-1.725199000	0.686620000
C	3.056989000	-0.743573000	0.099970000
C	3.627941000	0.430893000	-0.395007000
C	5.003570000	0.593233000	-0.293988000
C	5.834649000	-0.387806000	0.294465000
C	5.252420000	-1.554812000	0.788140000
H	3.421307000	-2.621269000	1.099424000
H	3.008614000	1.184270000	-0.881233000
H	5.866299000	-2.323460000	1.256439000
C	7.119384000	1.371608000	-0.416754000
C	8.257339000	2.136242000	-0.654701000
C	9.477402000	1.617944000	-0.243250000
C	9.565022000	0.370119000	0.388760000
C	8.426542000	-0.386542000	0.622283000
C	7.186756000	0.110512000	0.218769000
H	8.193154000	3.106309000	-1.145377000
H	10.384674000	2.193784000	-0.417580000
H	10.537501000	-0.006427000	0.699627000
H	8.498788000	-1.356714000	1.113072000
N	5.796522000	1.642382000	-0.717725000
H	5.462306000	2.477060000	-1.175293000
H	-1.778953000	1.348789000	1.281496000
C	-4.905143000	-1.924738000	-0.401672000
C	-5.313752000	-0.599811000	-0.141677000
C	-4.329890000	0.383482000	0.096608000
C	-2.999939000	0.026562000	0.067942000
C	-2.610112000	-1.302910000	-0.193585000
C	-3.569450000	-2.285416000	-0.428425000
H	-5.671057000	-2.678443000	-0.583595000
H	-4.614091000	1.413451000	0.300980000
H	-3.279245000	-3.315297000	-0.631483000
C	-1.781983000	0.884127000	0.284077000
H	-1.729401000	1.712635000	-0.438032000
C	-0.651341000	-0.093396000	0.111204000
C	0.712163000	0.117950000	0.199013000
C	1.599334000	-0.952505000	0.012990000
C	1.076153000	-2.228294000	-0.259182000
C	-0.289804000	-2.448416000	-0.348432000
C	-1.158013000	-1.375388000	-0.162350000
H	1.104764000	1.105724000	0.442449000
H	1.765883000	-3.053050000	-0.432492000
H	-0.672520000	-3.444496000	-0.569456000
C	-7.460961000	0.778012000	0.073850000
C	-6.907804000	2.055792000	0.375601000
C	-8.935619000	0.654291000	-0.013847000
N	-6.445140000	3.095281000	0.621618000
O	-9.530804000	-0.369717000	-0.260110000
O	-9.553076000	1.824504000	0.213483000
H	-10.506309000	1.645826000	0.135497000
C	-6.733943000	-0.350457000	-0.142616000
H	-7.360407000	-1.219657000	-0.357516000

Optimized Cartesian coordinates for the CFLA molecular system

Geometrical parameters

Center Number	X	Y	Z
C	-7.591622000	1.959832000	-0.718904000
C	-6.762738000	0.942030000	-0.199260000
C	-7.339143000	-0.188174000	0.387138000
C	-8.723941000	-0.271599000	0.439746000
C	-9.558480000	0.748608000	-0.070974000
C	-8.970407000	1.871519000	-0.653715000
H	-7.128894000	2.816771000	-1.204895000
H	-6.714557000	-0.971361000	0.817140000
H	-9.589940000	2.667590000	-1.065431000
C	-10.853784000	-0.925287000	0.805814000
C	-12.000559000	-1.620015000	1.177798000
C	-13.226905000	-1.035109000	0.892865000
C	-13.311986000	0.209332000	0.254117000
C	-12.164712000	0.896148000	-0.113675000
C	-10.918923000	0.331027000	0.160730000
H	-11.939106000	-2.587274000	1.674292000
H	-14.141033000	-1.556035000	1.172498000
H	-14.289900000	0.638865000	0.045791000
H	-12.232846000	1.863716000	-0.610142000
N	-9.522985000	-1.268736000	0.965121000
H	-9.187466000	-2.116677000	1.396466000
H	2.427879000	1.829632000	-0.290252000
C	4.571529000	-2.485627000	-0.687855000
C	5.268031000	-1.371419000	-0.188158000
C	4.548268000	-0.213101000	0.151482000
C	3.177836000	-0.188238000	-0.008231000
C	2.491894000	-1.311855000	-0.503832000
C	3.194597000	-2.465388000	-0.844412000
H	5.130360000	-3.372408000	-0.982608000
H	5.069081000	0.654299000	0.559719000
H	2.677383000	-3.339184000	-1.238993000
C	2.204126000	0.920521000	0.287993000
H	2.228775000	1.218184000	1.347174000
C	0.881572000	0.314175000	-0.098523000
C	-0.382975000	0.864992000	-0.035795000
C	-1.494103000	0.110630000	-0.448938000
C	-1.289735000	-1.201668000	-0.908771000
C	-0.022667000	-1.762167000	-0.962969000
C	1.070624000	-1.000824000	-0.559334000
H	-0.528541000	1.872342000	0.354444000
H	-2.145073000	-1.784372000	-1.250212000
H	0.107779000	-2.781098000	-1.326198000
C	-5.312813000	1.090011000	-0.280044000
C	-4.573593000	2.245300000	-0.349173000
C	-3.179841000	2.024597000	-0.414799000
C	-2.828484000	0.696792000	-0.398572000
S	-4.258697000	-0.300192000	-0.301917000
H	-5.026575000	3.233688000	-0.329466000
H	-2.448569000	2.825801000	-0.494333000
C	9.146417000	-0.900762000	0.209877000
C	8.867785000	-2.258519000	0.232801000
C	7.507913000	-2.558803000	0.097914000
C	6.714935000	-1.433099000	-0.032207000
S	7.670108000	0.017716000	0.001578000
H	9.651750000	-3.002227000	0.359451000
H	7.103375000	-3.566858000	0.127993000
C	10.453019000	-0.360783000	0.336462000
C	10.885314000	0.931668000	0.348689000
H	11.250037000	-1.101161000	0.442532000
C	10.005352000	2.042401000	0.227077000
N	9.258572000	2.930372000	0.126307000
C	12.336599000	1.162472000	0.498940000
O	13.166586000	0.288457000	0.610801000
O	12.642786000	2.470017000	0.498220000
H	13.609017000	2.520443000	0.600494000

Optimized Cartesian coordinates for the CIA molecular system

Geometrical parameters

Center Number	Coordinates (Angstroms) X	Y	Z
C	5.963027000	1.671588000	0.522704000
C	5.198756000	0.573106000	0.077684000
C	5.845643000	-0.600780000	-0.314952000
C	7.232697000	-0.646780000	-0.255702000
C	8.002248000	0.452983000	0.187067000
C	7.345289000	1.618777000	0.580125000
H	5.446312000	2.569185000	0.858809000
H	5.272455000	-1.447075000	-0.692931000
H	7.911745000	2.479065000	0.935401000
C	9.399150000	-1.270796000	-0.388004000
C	10.586340000	-1.966948000	-0.593865000
C	11.774382000	-1.313016000	-0.297625000
C	11.782642000	-0.000203000	0.192130000
C	10.595506000	0.687450000	0.395130000
C	9.386499000	0.054394000	0.104980000
H	10.584068000	-2.987585000	-0.973624000
H	12.719096000	-1.832338000	-0.449679000
H	12.732410000	0.482794000	0.413459000
H	10.605746000	1.708812000	0.774433000
H	0.603099000	-2.271073000	-0.043703000
C	-2.843085000	1.117437000	-0.220027000
C	-3.115077000	-0.206584000	0.179448000
C	-2.083829000	-1.101022000	0.470367000
C	-0.782807000	-0.646377000	0.353276000
C	-0.509714000	0.678315000	-0.048635000
C	-1.540305000	1.572103000	-0.337547000
H	-2.297998000	-2.125258000	0.778846000
H	-1.325888000	2.596090000	-0.646012000
C	0.506615000	-1.383662000	0.600393000
H	0.579742000	-1.749362000	1.636048000
C	1.552456000	-0.345970000	0.293320000
C	2.930133000	-0.445988000	0.350043000
C	3.726814000	0.662704000	0.027090000
C	3.096148000	1.859640000	-0.349120000
C	1.713761000	1.967561000	-0.406311000
C	0.936545000	0.859384000	-0.082835000
H	3.404164000	-1.373560000	0.673769000
H	3.711890000	2.713148000	-0.629802000
H	1.249868000	2.905920000	-0.709827000
C	-4.559036000	-0.389455000	0.207467000
C	-4.131237000	1.856008000	-0.466668000
C	-5.176300000	0.815442000	-0.171100000
H	-4.203097000	2.231014000	-1.498811000
H	-4.234445000	2.736752000	0.185131000
C	-5.335859000	-1.497201000	0.542133000
C	-6.551965000	0.917142000	-0.224951000
C	-6.715758000	-1.394729000	0.481559000
C	-7.343213000	-0.200918000	0.087266000
H	-7.026768000	1.854656000	-0.514767000
H	-4.871631000	-2.429492000	0.861463000
H	-7.322257000	-2.248936000	0.776648000
C	-8.812589000	-0.117682000	-0.004554000
C	-9.606854000	-1.201782000	-0.152092000
C	-9.345453000	1.216774000	0.053387000
C	-11.072681000	-1.261749000	-0.223712000
O	-11.677135000	-2.300266000	-0.372756000
O	-11.681590000	-0.070456000	-0.109287000
N	-9.661828000	2.334093000	0.097163000
H	-9.148945000	-2.182790000	-0.251247000
H	-12.636309000	-0.246334000	-0.175559000
N	8.092260000	-1.673795000	-0.597999000
H	7.810187000	-2.574936000	-0.953078000

Optimized Cartesian coordinates for the CSILAA molecular system

Geometrical parameters

Center Number	Coordinates (Angstroms) X	Y	Z
C	-3.893563000	1.652560000	0.701584000
C	-3.223830000	0.536717000	0.158616000
C	-3.964530000	-0.532767000	-0.349735000
C	-5.350524000	-0.459611000	-0.305143000
C	-6.026628000	0.657823000	0.236851000
C	-5.275879000	1.717649000	0.744891000
H	-3.305456000	2.464390000	1.126866000
H	-3.462810000	-1.386212000	-0.805172000
H	-5.769466000	2.586067000	1.179836000
C	-7.560557000	-0.872291000	-0.508564000
C	-8.800557000	-1.439756000	-0.785422000
C	-9.931033000	-0.715245000	-0.434059000
C	-9.832096000	0.541673000	0.177817000
C	-8.592694000	1.100684000	0.451131000
C	-7.439404000	0.393896000	0.108513000
H	-8.882247000	-2.416351000	-1.260269000
H	-10.914812000	-1.134649000	-0.637632000
H	-10.738844000	1.083074000	0.440246000
H	-8.518829000	2.078359000	0.926531000
N	-6.291712000	-1.368075000	-0.749452000
H	-6.086050000	-2.253670000	-1.186670000
C	4.802418000	1.056851000	-0.215690000
C	5.297949000	-0.239447000	0.022327000
C	4.369758000	-1.272249000	0.259683000
C	3.007021000	-1.037247000	0.262580000
C	2.530025000	0.272395000	0.019418000
C	3.442135000	1.305819000	-0.216833000
H	5.489134000	1.878321000	-0.402581000
H	4.754490000	-2.277097000	0.443612000
H	3.092322000	2.319899000	-0.404564000
C	0.330932000	-0.751056000	0.298185000
C	-1.054640000	-0.702732000	0.339399000
C	-1.750383000	0.496116000	0.125120000
C	-1.002610000	1.654184000	-0.127072000
C	0.382997000	1.626852000	-0.168884000
C	1.065553000	0.428129000	0.040370000
H	-1.625774000	-1.603099000	0.570072000
H	-1.524154000	2.590174000	-0.323195000
H	0.926604000	2.547649000	-0.377719000
Si	1.534976000	-2.164370000	0.544603000
H	1.410197000	-3.249149000	-0.458490000
H	1.495679000	-2.770065000	1.896828000
C	6.694757000	-0.599580000	0.041084000
C	7.827036000	0.129381000	-0.139055000
C	7.856989000	1.528874000	-0.404143000
C	9.116044000	-0.598996000	-0.048313000
H	6.899215000	-1.655464000	0.235870000
N	7.866130000	2.672777000	-0.619322000
O	9.225978000	-1.783436000	0.170497000
O	10.166854000	0.213478000	-0.239354000
H	10.959444000	-0.345990000	-0.163670000

Optimized Cartesian coordinates for the CSILOA molecular system

Geometrical parameters

Center Number	Coordinates (Angstroms) X	Y	Z
C	-3.768385000	1.796698000	-0.547450000
C	-2.839516000	0.837618000	-0.087680000
C	-3.285764000	-0.427363000	0.307134000
C	-4.643698000	-0.701009000	0.231252000
C	-5.580566000	0.257987000	-0.220157000
C	-5.121305000	1.516800000	-0.610238000
H	-3.401757000	2.763214000	-0.888203000
H	-2.587194000	-1.170019000	0.692677000
H	-5.818697000	2.270045000	-0.974853000
H	-4.897839000	-2.703473000	0.907826000
C	-6.675020000	-1.677695000	0.329938000
C	-7.729056000	-2.567472000	0.512013000
C	-9.004859000	-2.123411000	0.195004000
C	-9.228048000	-0.827339000	-0.290596000
C	-8.173015000	0.054052000	-0.469278000
C	-6.878777000	-0.366445000	-0.158786000
H	-7.559682000	-3.574649000	0.889505000
H	-9.849121000	-2.797808000	0.326473000
H	-10.241936000	-0.512999000	-0.529915000
H	-8.350061000	1.059942000	-0.848687000
N	-5.322736000	-1.856733000	0.560431000
C	3.250280000	2.112617000	0.187140000
C	2.486564000	0.952514000	0.035210000
C	1.056545000	1.115966000	0.011537000
C	0.562182000	2.406449000	0.141795000
C	-1.423517000	1.181556000	-0.029691000
C	-0.848131000	2.426844000	0.115511000
H	2.104344000	4.258595000	1.610167000
H	2.172465000	4.529349000	-0.785445000
H	-1.450568000	3.324749000	0.232875000
S	3.423062000	-0.485003000	-0.091657000
S	-0.196325000	-0.062700000	-0.153907000
C	4.615188000	1.811030000	0.200228000
C	4.905388000	0.457064000	0.061771000
H	5.413344000	2.543442000	0.309085000
Si	2.045170000	3.546530000	0.314011000
C	6.219143000	-0.072970000	0.050078000
C	6.660861000	-1.358032000	-0.071575000
H	7.015679000	0.667569000	0.158410000
C	5.779871000	-2.464665000	-0.218417000
C	8.119125000	-1.581533000	-0.044983000
N	5.033942000	-3.351576000	-0.336639000
O	8.949612000	-0.708524000	0.074792000
O	8.434581000	-2.881208000	-0.170975000
H	9.405930000	-2.925691000	-0.142693000

Ultraviolet-Visible absorption spectra

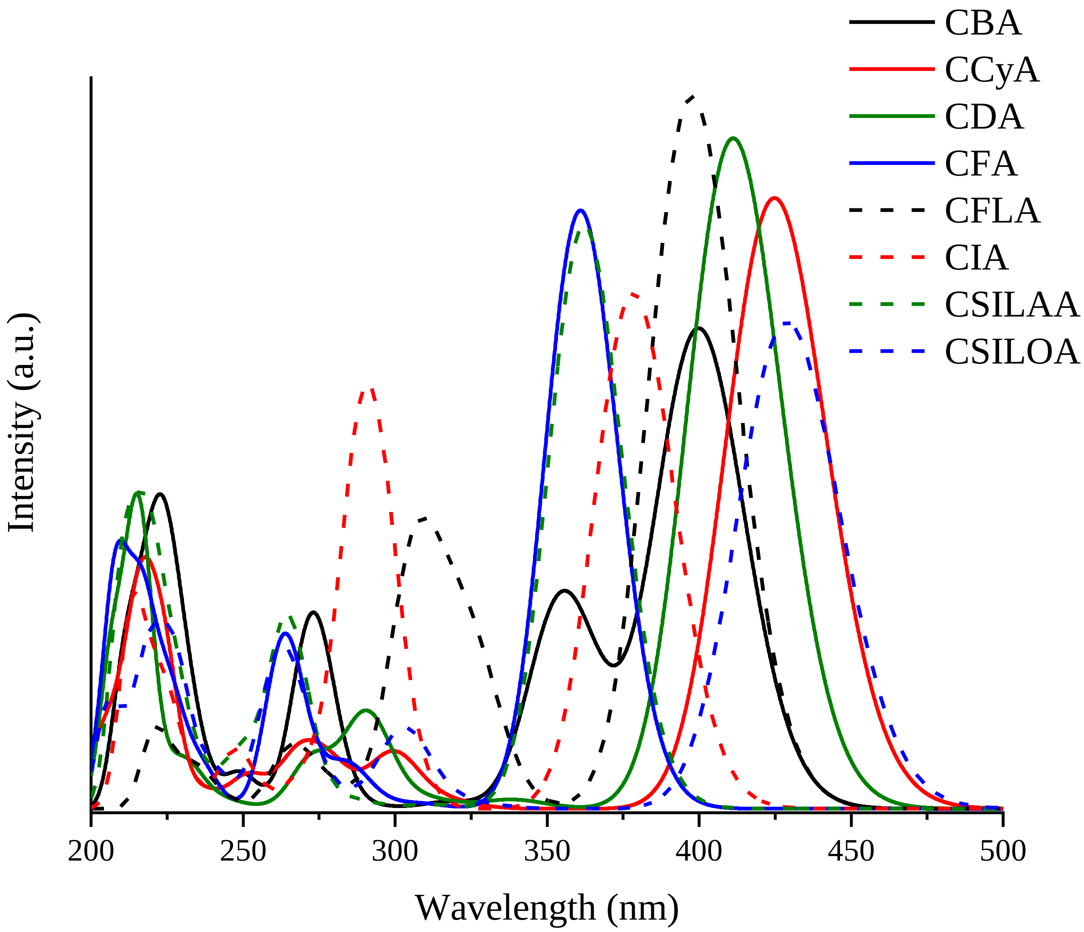

Figure S1. UV-Vis absorption spectra of carbazole-based dyes with the CAM-B3LYP/6-31G(d) level of theory.

Chemical reactivity parameters

Table S1. Chemical reactivity parameters of carbazole-based dyes (in eV) obtained by DFT conceptual at CAM-B3LYP/6-31G(d) level of theory.

**Molecules**	η	ω	ω^{-}	ω^{+}
CBA	5.75	1.53	5.52	1.32
CCyA	5.57	1.43	5.21	1.22
CDA	5.64	1.37	5.05	1.12
CFA	6.33	1.39	5.26	1.07
CFLA	5.54	1.41	5.15	1.19
CIA	5.92	1.30	4.94	1.01
CSILAA	6.26	1.45	5.43	1.17
CSILOA	5.57	1.53	5.48	1.35
